# Inhaled colistin for the treatment of ventilator-associated tracheobronchitis in critically ill patients

**DOI:** 10.1186/cc9987

**Published:** 2011-03-11

**Authors:** Z Athanassa, P Myrianthefs, E Boutzouka, V Papaioannou, S Tsiplakou, A Tsakris, G Baltopoulos

**Affiliations:** 1 'Agioi Anargyroi' Hospital, Athens, Greece; 2 'KAT' Hospital, Athens, Greece; 3 Medical School, University of Athens, Greece

## Introduction

Limited evidence exists regarding the efficacy of inhaled antibiotics in ventilator-associated tracheobronchitis (VAT) [1,2]. The aim of this study was to assess the effect of monotherapy with nebulized colistin on clinical and microbiological outcomes in critically ill patients with VAT due to polymyxin-only susceptible Gram-negative bacteria.

## Methods

Patients were eligible for the study if they had clinical symptoms suggestive of VAT (for example, fever, purulent secretions), an absence of an evolving infiltrate on chest X-ray, and microbiological confirmation of VAT with quantitative cultures of endotracheal aspirates (ETAs) with a diagnostic threshold for VAT ≥10^5 ^colony-forming units (CFU)/ml. Susceptibility to colistin was determined using the Vitek technique (Biomerieux, France). Selected patients received inhaled colistin at a dose of 1 million units every 8 hours for 7 days via a vibrating-mesh nebulizer (Aeroneb Pro; Aerogen, Galway, Ireland). Assessed clinical outcomes were cure, defined as resolution of signs and symptoms at day 5, and development of ventilator-associated pneumonia (VAP) at day 10 after initiation of treatment. Microbiological outcomes were defined as eradication and decline termed as isolation of ≤10^2 ^CFU/ml and were assessed by ETA quantitative cultures received at days 3 and 5 after initiation of treatment.

## Results

Our study included 12 patients (eight men and four women) with mean age 58.7 years. The mean values of APACHE II and SOFA scores were 15.5 and 6.8, respectively. Two patients had polymicrobial Gram-negative VAT. Isolated pathogens from ETAs were: *Pseudomonas aeruginosa *(8/12 patients), *Acinetobacter baumannii *(5/12 patients), and *Klebsiella pneumoniae *(1/12 patients). Cure was achieved in nine out of 12 patients. In the three patients with clinical failure, intravenous colistin was administered. Two of them were subsequently cured and one patient developed VAP. Microbiological eradication was achieved in five out of 12 patients while decline was achieved in three out of 12 patients.

## Conclusions

According to our limited data, monotherapy with nebulized colistin might be effective in the treatment of patients with VAT. Further investigation is warranted to evaluate whether nebulized antibiotics could effectively treat VAT and reduce the need for systemic antibiotics.
